# Validation of Type 2 Diabetes Risk Variants Identified by Genome-Wide Association Studies in Northern Han Chinese

**DOI:** 10.3390/ijerph13090863

**Published:** 2016-08-30

**Authors:** Ping Rao, Yong Zhou, Si-Qi Ge, An-Xin Wang, Xin-Wei Yu, Mohamed Ali Alzain, Andrea Katherine Veronica, Jing Qiu, Man-Shu Song, Jie Zhang, Hao Wang, Hong-Hong Fang, Qing Gao, You-Xin Wang, Wei Wang

**Affiliations:** 1 Beijing Key Laboratory of Clinical Epidemiology, School of Public Health, Capital Medical University, Beijing 100069, China; raopingbj@126.com (P.R.); gesky90@sina.com (S.-Q.G.); yuxinweigongzuo@163.com (X.-W.Y.); mohali@163.com (M.A.A.); andrea_eleanor@hotmail.com (A.K.V.); songms@ccmu.edu.cn (M.-S.S.); zhangjie@ccmu.edu.cn (J.Z.); wanghaostudy@163.com (H.W.); fanghonghong79@sina.com (H.-H.F.); shouyigaoqing@163.com (Q.G.); wei.wang@ecu.edu.au (W.W.); 2 Beijing Rehabilitation Hospital, Capital Medical University, Beijing 100144, China; 3 Beijing Institute of Heart, Lung and Blood Vessel Diseases, Beijing Anzhen Hospital, Capital Medical University, Beijing 100029, China; yongzhou78214@163.com; 4 School of Medical Sciences, Edith Cowan University, Perth, WA 6027, Australia; 5 Department of Neurology, Beijing Tiantan Hospital, Capital Medical University, Beijing 100050, China; anxin0907@163.com; 6 School of Public Health, Ningxia Medical University, Yinchuan 750021, China; qiu199008jing@163.com

**Keywords:** type 2 diabetes, genetic susceptibility, *FAF1*, genetic risk score, Han Chinese

## Abstract

*Background*: More than 60 genetic susceptibility loci associated with type 2 diabetes mellitus (T2DM) have been established in populations of Asian and European ancestry. Given ethnic differences and environmental factors, validation of the effects of genetic risk variants with reported associations identified by Genome-Wide Association Studies (GWASs) is essential. The study aims at evaluating the associations of T2DM with 29 single nucleotide polymorphisms (SNPs) from 19 candidate genes derived from GWASs in a northern Han Chinese population. *Method*: In this case-control study, 461 T2DM-diagnosed patients and 434 controls were recruited at the *Jidong* oil field hospital (Hebei, China) from January 2009 to October 2013. A cumulative genetic risk score (cGRS) was calculated by summation of the number of risk alleles, and a weight GRS (wGRS) was calculated as the sum of risk alleles at each locus multiplied by their effect sizes for T2DM, using the independent variants selected. *Result*: The allelic frequency of the “A” allele at rs17106184 (Fas-associated factor 1, *FAF1*) was significantly higher in the T2DM patients than that of the healthy controls (11.7% vs. 6.4%, *p* < 0.001). Individuals in the highestquartile of wGRS had an over three-fold increased risk for developing T2DM compared with those in the lowest quartile (odds ratio = 3.06, 95% CI = 1.92–4.88, *p* < 0.001) adjusted for age, sex, BMI, total cholesterol (TC), triglycerides (TG), low-density lipoprotein cholesterol (LDL-C), systolic blood pressure (SBP) and diastolic blood pressure (DBP). The results were similar when analyzed with the cGRS. *Conclusions*: We confirmed the association between rs17106184 (*FAF1*) and T2DM in a northern Han Chinese population. The GRS calculated based on T2DM susceptibility variants may be a useful tool for predicting the T2DM susceptibility.

## 1. Introduction

Type 2 diabetes mellitus (T2DM) has been identified as a major international health challenge which has a great worldwide impact on morbidity, premature mortality, and economic burden [1]. In China, the prevalence of diabetes increased from 0.9% in 1980 to 11.6% in 2013, indicating that there were approximately 113.9 million Chinese adults suffering from diabetes [2]. T2DM is a complex metabolic syndrome with a strong genetic component contributing to its pathogenesis [3,4]. Candidate gene association studies and genome-wide association studies (GWASs) have facilitated the identification of genetic susceptibility variants for T2DM. To date, more than 60 susceptibility loci of T2DM have been identified in Asian and European populations [5,6,7,8,9,10,11,12,13,14]. 

The contributions of known single nucleotide polymorphisms (SNPs) in T2DM susceptibility genes are different among various populations. SNP rs864745 in juxtaposed with a zinc finger 1 (*JAZF1*) was identified as a T2DM susceptibility locus in a European population by GWAS [5] and also in Japanese [8], Han Chinese [11], and Lebanese Arab ethnic groups [15]. However, the association is inconsistent in terms of risk allele frequencies and odds ratios (ORs) among these populations. Another fat mass and obesity associated SNP rs8050136 (*FTO*) was associated with T2DM in UK samples [7], whereas it was not found to be associated with T2DM in Chinese samples [16]. When examined individually, each of the genetic susceptibility loci only confers a small-to-moderate disease risk, and thus is of limited utility in risk prediction of T2DM [17]. Combining multiple T2DM-related loci with modest effects using a genetic risk score (GRS) may be useful in the risk stratification of T2DM [18,19]. 

In this case-control study, we investigated the associations between 29 SNPs susceptibility loci and T2DM, and the combined effects of these independent SNPs on the risk of T2DM in a northern Han Chinese population. 

## 2. Experimental Section

### 2.1. Study Participants

In this case-control study, 461 T2DM-diagnosed patients and 434 controls were recruited at the *Jidong* oil field hospital (Hebei, China) from January 2009 to October 2013. The participants in the study were the same as previously reported [20]. In brief, T2DM patients were diagnosed and confirmed according to the American Diabetes Association criteria [21] or had documented clinical diagnosis of T2DM from clinical records. Written informed consent was obtained from each participant before beginning participation in the study. The study was approved by the Ethics Committee of Capital Medical University, Beijing, China (approval number 2013SY30).

### 2.2. Selection of SNPs and Genotyping

The minor allele frequency (MAF) of these selected SNPs was more than 0.05 in HapMap-CHB data [22]. Twenty-nine genetic risk SNPs were selected from GWASs or well-established association studies for T2DM in European or Asian populations. The associations between rs4402960, rs1470579, and susceptibility to T2DM in this population have been reported in Rao et al. [20].

A blood sample from each participant was drawn into a 5 mL vacuum tube with ethylene diamine tetraacetic acid (EDTA). All the samples were centrifuged at 4000× *g* for 5 min to separate the plasma content. Genomic DNA was extracted from peripheral white blood cells using blood genome DNA extraction kits, according to the manual instructions (BioTeke, Beijing, China). DNA samples were stored at −80 °C before usage. SNPs were genotyped using Mass ARRAY system (Sequenom, Inc., San Diego, CA, USA). DNA from patients and controls were randomly assigned to 96-well plates and genotyped using a blinded method. The call rates for the genotyping of the SNPs were >98%.

### 2.3. Data Collection

Data were collected by a comprehensive review of hospital records. Hospital records included information on overnight fasting measurements of fasting plasma glucose (FPG), triglycerides (TG), total cholesterol (TC), low-density lipoprotein cholesterol (LDL-C), systolic blood pressure (SBP), and diastolic blood pressure (DBP) tested by standard methods in the clinical laboratory of the *Jidong* oil field hospital [21]. Weight and height were measured when the participants were lightly clothed and barefoot. Body mass index (BMI) was calculated as weight in kilograms divided by the square of height in meters (kg/m^2^), and was classified as normal (<24 kg/m^2^), overweight (24 to 28 kg/m^2^), or obese (≥28 kg/m^2^) [23]. 

### 2.4. Statistical Analysis

Analyses were conducted with SPSS Software V.18.0 (IBM, Chicago, IL, USA). *Chi*-square test was used to test Hardy-Weinberg equilibrium (HWE) for genotype frequencies. Continuous variables were presented as mean ± standard deviation (SD). Categorical variables were presented as numbers and percentages. Student’s *t* test was used to test between-group differences for continuous variables. *Chi*-square test was applied for categorical variables. After excluding one SNP due to its deviation from HWE, associations between 28 SNPs and T2DM risks were assessed using ORs with 95% CIs and *p* value derived from unconditional logistic regression (ULR) analyses adjusted for age, sex, BMI, TG, TC, LDL-C, SBP, and DBP. Bonferroni correction was used in the association analysis when multiple comparisons were carried out. The statistical powers for the association between rs17106184 and T2DM in different genetic models were estimated using Quanto version 1.2.4 (University of Southern California, Los Angeles, CA, USA). 

Linkage disequilibrium analysis wasperformed when more than one candidate SNP in a gene wasselected, and only independent variants in each gene (with the highest OR in association analysis) were used to construct genetic risk scores. Two types of genetic risk score (GRS) were constructed based on the independent SNPs. First, the cumulative genetic risk score (cGRS) was determined by a simple summation of the number of risk alleles from the SNPs based on the previous studies. Second, the weighted genetic risk score (wGRS) was calculated using the beta-coefficients of a logistic regression model, according to a method reported by Chang et al. [16]. All participants were divided into four equal groups according to their wGRS or cGRS. The OR and 95% CI for each group were estimated using the lowest quartile group as the reference group derived from ULR. The significance level was set at *p* < 0.05 (two-tailed).

## 3. Results

### 3.1. Characteristics of the Participants and SNP Information

The demographic information and clinical characteristics of the participants can be found in our previous report [20]. In total, 274 male and 187 female (53.48 ± 11.33 years) patients, and 249 male and 185 female (51.82 ± 12.67 years) controls were included in the final analysis.

Among the 29 SNPs tested, 28 were consistent with HWE (*p* > 0.05), except for rs1111875 (*HHEX*) (*p* = 0.008) in the control, and thus this SNP was excluded in the further analysis. The MAF of these SNPs ranged from 0.09 to 0.49. The basic information of these SNPs is summarized in Table 1.

### 3.2. Association Analysis of the Candidate SNPs for T2DM

Twenty-eight candidate SNPs were selected for further association analysis, and thus the significance level was adjusted to 0.0018 (0.05/28). Of the SNPs genotyped, rs17106184 in Fas-associated factor 1 (*FAF1*) was significantly associated with T2DM, even after adjusting for age, sex, BMI, TC, TG, LDL-C, SBP, and DBP (adjusted odds ratio (AOR) = 2.22, 95% CI = 1.53–3.24, *p* < 0.0001) (Table 2). There was no statistically significant association of the variants in the remaining 27 SNPs with T2DM in the 18 genes studied (*p* > 0.05).

In the dominant model (AA + AG vs. GG), the carriers of AA + AG at rs17106184 had a higher risk of T2DM compared to the carriers of GG (AOR = 2.32, 95% CI = 1.57–3.47, *p* = 4.61 × 10^−5^). In the additive model, the carriers of AG were more susceptible to T2DM than the carriers of GG (AOR = 2.14, 95% CI = 1.47–3.12, *p* = 7.96 × 10^−5^). There was no statistical significance found in recessive model of the “A” allele (*p* = 0.17, Table 3). The statistical powers were estimated to be 0.18, 0.99, and 0.99 in recessive, dominant, and additive models, respectively.

### 3.3. Genetic Risk Score and Diabetes Risk

**Figure 1:**
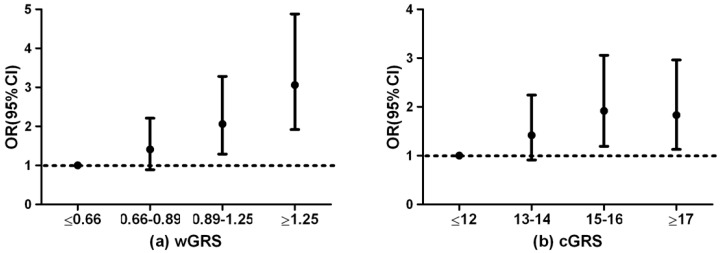
Odds ratio (OR) of T2DM according to the wGRS (**a**) and cGRS (**b**).

To investigate the cumulative effect of the risk alleles on T2DM risk, wGRS and cGRS were calculated for all participants. Four variants in*SLC16A11*, three in *CDKAL1*, two in *C2CD4A/B*, and two in *IGF2BP2* were in strong linkage disequilibrium (*r*^2^ range from 0.78 to 1.00, Figure S1); therefore four variants in each gene with the highest OR in association analysis together with another 15 variants in 15 genes were used to construct genetic risk scores. The median wGRS was 0.98 (interquartile range (IQR): 0.71) and 0.83 (IQR: 0.46) in T2DM and controls, respectively, while the median cGRS was 15 (IQR: 4) and 14 (IQR: 4) in T2DM and controls, respectively. Both wGRS and cGRS were significantly associated with T2DM susceptibility (AOR = 1.93, 95% CI = 1.42–2.62, *p* < 0.001; AOR = 1.07, 95% CI = 1.01–1.13, *p* = 0.030, respectively). In addition, the individuals in the highest quartile of the wGRS had a nearly three-fold increased risk of developing T2DM compared with the lowest quartile (AOR = 3.06, 95% CI = 1.92–4.88, *p* < 0.001). Similarly, the individuals with 17 or more risk alleles among the 19 loci tested were more likely to have T2DM than those with 0–12 risk alleles among the loci tested (AOR = 1.83, 95% CI = 1.13–2.96, *p* = 0.010, Figure 1).

## 4. Discussion

We examined the associations of T2DM with 28 SNPs from 19 candidate genes derived from GWASs in a northern Han Chinese population. Of the studied variants, rs17106184 (*FAF1*) was significantly associated with T2DM. There was a significant difference in the frequency of the “A” allele at rs17106184 between the T2DM patients and the healthy controls (*p* < 0.0001). In addition, we found that the GRS calculated based on the 28 genetic variants genotyped was significantly associated with T2DM susceptibility (*p* < 0.05). 

To our knowledge, this is the first study reporting that the “A” allele of rs17106184 (*FAF1*) is associated with T2DM susceptibility in a northern Han Chinese population. In contrast, the G allele at this same locus was associated with increased T2DM risk in a European population [13]. Three explanations might address on this inconsistency between the two findings. Firstly, ethnic differences and disease heterogeneity might exist among the study subjects. Secondly, rs17106184 may be a proxy marker rather than a true functional variant, so that the two different risk alleles in different ethnic populations are in Linkage disequilibrium (LD) with the unidentified causative gene allele, warranting more validation studies in different ethnic groups to investigate the association of rs17106184 with T2DM. Thirdly, the limited sample size might be underpowered to detect a direction of the association.

Chronic low-grade inflammation plays an important role in the pathogenesis of T2DM [24]. Epidemiology investigations have revealed that certain genetic variants related to inflammation are associated with a risk for T2DM [25,26]. *FAF1* (1p33), expressed in the cardiac muscles, testes, skeletal muscles, pancreas, and some other tissues [27] can robustly suppress NF-κB activation by disrupting IêB kinase (IKK) complex assembly and preventing nuclear translocation of NF-κB RelA (p65) in a stimulation-dependent manner [28]. Activation of the NF-κB signaling pathway (which is closely correlated with inflammation) has been implicated in the pathogenesis of impaired insulin secretion, insulin resistance, and diabetic vascular complications in T2DM [29,30]. SNP rs17106184 locates in the 18th intron of *FAF1*, which might alter the splicing of primary transcripts or gene expression [31]. Association of rs17106184 with T2DM suggests that rs17106184 may affect NF-κB activation and, therefore, decrease insulin translation and sensitivity, which leads to T2DM.

We also constructed the LD pattern of a 100-kb 1p33 region based on HapMap-CHB data. Rs7525764, rs2055491, and rs17106184 were located in a 73kb haplotype block (Figure S2). In a European group, rs7525764 and rs2055491 are associated with ulcerative colitis (UC) (*p* = 0.0192; *p* = 0.0009, respectively) [32]. Furthermore, the risk allele “G” of rs7525764 and “T” of rs2055491 for UC co-occur with the “A” allele of rs17106184 in the same haplotype. The “A” allele of rs17106184 may be able to escalate the inflammatory response, which plays a role in the pathogenesis of UC [33]. Considering that inflammation is involved in the common pathogenesis of chronic disease, including T2DM, hypertension, and UC [34], it can be inferred that the “A” allele of rs17106184 is associated with the pathogenesis of T2DM via inflammation pathway.

The previous studies showed that accumulative number of risk alleles may be associated with T2DM, although these alleles were not observed to be statistically significant individually in the association study [16,35]. GRS associated with T2DM could be used as a simple proxy of the contribution each of individual genetic locus to the predisposition to T2DM [19,36]. For example, compared with participants in the lowest quintile of GRS, calculated on the basis of 10 polymorphisms in 9 genes, men in the highest quintile have an OR for T2DM of 2.76 (95% CI = 2.06–3.68), and women in this quintile have an OR of 2.17 (95% CI = 1.76–2.69), while the per-allele OR for the risk of T2DM is 1.19 and 1.16 for men and women, respectively, in a European study [37]. In a cross-sectional case-control study of 2613 T2DM cases and 1786 controls from a Japanese population, a GRS-49 was calculated based on 49 SNPs. Those with a GRS-49 > 60 are 9.81 times more likely to have T2DM than those with a GRS-49 < 46, and the OR per risk allele for the development of T2DM is 1.13 (95% CI = 1.11–1.15) [36]. In this study, we found that the OR of T2DM in the subjects with the highest wGRS quartile was 3.06 compared with those in the lowest wGRS quartile (AOR = 3.06, 95% CI = 1.92–4.88, *p* < 0.001). This result was consistent with Chauhan’s study [38]. 

Limitations of the study: Firstly, the present study is based on a case-control design, lacking the power of causal inference. The underlying pathogenesis of *FAF1* should be identified in a further functional study. Secondly, some of the 28 SNPs are in strong linkage disequilibrium, which may make double or more effects when calculating GRS. However, some scholars do not support the exclusion of these SNPs in calculating GRS [16,19,39]. Thirdly, the occurrence of diabetes depends on the interactions between the presence of different risk alleles and environmental factors. The influence of a single polymorphism is rather small and may not be directly linked to the true causal variants. Incorporation of new genetic variants and environmental factors should be included in further research to enhance the predictions. 

## 5. Conclusions

This study suggests that gene polymorphisms of *FAF1* (rs17106184) are associated with a risk of T2DM in a northern Han Chinese population. The GRS we constructed by accumulating the power of 19 susceptibility SNPs in 19 genes makes it a possible tool to identify individuals with a high risk of developing T2DM.

## Figures and Tables

**Table 1:** Information of the selected type 2 diabetes mellitus (T2DM)-related single nucleotide polymorphisms (SNPs).

No.	SNP rs#	Gene	Chr.	Chr. Position	Risk/Non-RiskAllele	HW-P		Call Rate
						Case	Control	
1	rs17106184	*FAF1*	1	50444313	A/G	1.00	0.24	98.2
2	rs780094	*GCKR*	2	27518370	A/G	0.78	1.00	98.9
3	rs3773159	*MGLL*	3	127720095	T/C	1.00	0.79	98.9
4	rs4402960	*IGF2BP2*	3	185793899	T/G	0.21	0.09	98.1
5	rs1470579	*IGF2BP2*	3	185811292	C/A	0.19	0.05	98.1
6	rs702634	*ARL15*	5	53975590	A/G	1.00	0.81	99.1
7	rs4712523	*CDKAL1*	6	20657333	A/G	0.50	0.2	98.5
8	rs4712524	*CDKAL1*	6	20657634	A/G	0.85	1.00	98.2
9	rs10946398	*CDKAL1*	6	20660803	A/C	0.70	0.38	98.6
10	rs7756992	*CDKAL1*	6	20679478	A/G	0.09	1.00	98.1
11	rs3130501	*POU5F1-TCF19*	6	31168676	G/A	0.50	0.18	99.4
12	rs9472138	*VEGFA*	6	43844025	C/T	0.02	0.04	99.6
13	rs864745	*JAZF1*	7	28140937	G/A	0.48	0.89	99.0
14	rs13266634	*SLC30A8*	8	117172544	T/C	0.05	0.49	98.0
15	rs10811661	*CDKN2B*	9	22134095	T/C	0.57	0.29	98.8
16	rs12779790	*CDC123/CAMKID*	10	12286011	A/G	0.71	0.47	99.3
17	rs1111875	*HHEX*	10	92703125	A/G	0.57	0.008	99.0
18	rs7923837	*HHEX*	10	92722160	G/A	0.17	0.58	98.6
19	rs7903146	*TCF7L2*	10	112998590	T/C	1.00	1.00	98.6
20	rs1153188	*DCD*	12	54705212	A/T	1.00	1.00	99.0
21	rs1370176	*C2CD4A/B*	15	62105035	T/C	1.00	0.91	98.8
22	rs1436953	*C2CD4A/B*	15	62121815	A/G	0.84	0.83	98.8
23	rs8050136	*FTO*	16	53782363	A/C	0.83	0.07	97.8
24	rs7192960	*MAF/WWOX*	16	79382666	T/C	1.00	1.00	99.8
25	rs75493593	*SLC16A11*	17	7041768	T/G	1.00	0.82	98.5
26	rs75418188	*SLC16A11*	17	7042164	T/C	1.00	0.66	98.5
27	rs13342232	*SLC16A11*	17	7042621	G/A	1.00	0.66	98.0
28	rs13342692	*SLC16A11*	17	7042968	C/T	1.00	1.00	99.0
29	rs117767867	*SLC16A11*	17	7043011	T/C	1.00	1.00	99.0

Chr., chromosome; HW-P, *p*-value for Hardy-Weinberg equilibrium.

**Table 2:** Association of selected T2DM-related SNPs with T2DM risk.

No.	SNP rs#	Gene	Frequency of Risk Allele		Crude Model		Adjusted Model *	
			Case	Control	OR (95% CI)	*p*	OR (95% CI)	*p*
1	rs17106184	*FAF1*	0.12	0.06	1.91 (1.36–2.69)	<0.0001	2.22 (1.53–3.24)	<0.0001
2	rs780094	*GCKR*	0.53	0.52	0.96 (0.80–1.17)	0.71	0.95 (0.77–1.17)	0.60
3	rs3773159	*MGLL*	0.12	0.10	1.16 (0.86–1.56)	0.33	1.04 (0.75–1.45)	0.82
4	rs4402960	*IGF2BP2*	0.25	0.25	1.02 (0.82–1.26)	0.89	1.05 (0.83–1.34)	0.67
5	rs1470579	*IGF2BP2*	0.27	0.26	1.04 (0.84–1.28)	0.75	1.07 (0.85–1.36)	0.55
6	rs702634	*ARL15*	0.90	0.89	0.89 (0.66–1.20)	0.45	0.77 (0.55–1.08)	0.13
7	rs4712523	*CDKAL1*	0.57	0.55	0.92 (0.77–1.11)	0.41	0.84 (0.69–1.04)	0.11
8	rs4712524	*CDKAL1*	0.57	0.55	0.91 (0.75–1.11)	0.33	0.84 (0.68–1.03)	0.10
9	rs10946398	*CDKAL1*	0.57	0.56	0.94 (0.78–1.14)	0.55	0.87 (0.71–1.07)	0.18
10	rs7756992	*CDKAL1*	0.49	0.48	1.06 (0.88–1.28)	0.55	1.13 (0.92–1.39)	0.26
11	rs3130501	*POU5F1-TCF19*	0.70	0.68	0.91 (0.74–1.11)	0.35	0.81 (0.64–1.01)	0.06
12	rs9472138	*VEGFA*	0.91	0.90	0.88 (0.64–1.20)	0.41	0.75 (0.53–1.07)	0.11
13	rs864745	*JAZF1*	0.27	0.24	1.17 (0.94–1.45)	0.16	1.13 (0.89–1.43)	0.32
14	rs13266634	*SLC30A8*	0.42	0.42	1.02 (0.85–1.24)	0.81	0.96 (0.78–1.18)	0.68
15	rs10811661	*CDKN2B*	0.54	0.52	0.91 (0.75–1.09)	0.31	0.88 (0.71–1.08)	0.21
16	rs12779790	*CDC123/CAMKID*	0.85	0.84	0.89 (0.69–1.16)	0.39	0.83 (0.62–1.11)	0.20
17	rs7923837	*HHEX*	0.78	0.77	0.96 (0.76–1.20)	0.69	1.01 (0.79–1.30)	0.91
18	rs7903146	*TCF7L2*	0.09	0.09	1.01 (0.63–1.62)	0.97	1.05 (0.63–1.74)	0.86
19	rs1153188	*DCD*	0.09	0.09	1.21 (0.60–2.45)	0.60	1.32 (0.58–2.96)	0.51
20	rs1370176	*C2CD4A/B*	0.30	0.29	1.02 (0.83–1.25)	0.88	0.98 (0.78–1.23)	0.87
21	rs1436953	*C2CD4A/B*	0.36	0.35	1.06 (0.87–1.28)	0.59	1.02 (0.82–1.27)	0.85
22	rs8050136	*FTO*	0.13	0.11	1.26 (0.94–1.69)	0.12	1.16 (0.84–1.60)	0.37
23	rs7192960	*MAF/WWOX*	0.29	0.29	1.06 (0.82–1.24)	0.97	1.07 (0.85–1.34)	0.59
24	rs75493593	*SLC16A11*	0.13	0.12	1.13 (0.85–1.49)	0.41	1.14 (0.84–1.56)	0.39
25	rs75418188	*SLC16A11*	0.13	0.12	1.11 (0.84–1.47)	0.46	1.23 (0.83–1.53)	0.45
26	rs13342232	*SLC16A11*	0.14	0.12	1.11 (0.83–1.45)	0.51	1.11 (0.82–1.52)	0.49
27	rs13342692	*SLC16A11*	0.13	0.13	1.08 (0.82–1.43)	0.58	1.09 (0.80–1.48)	0.58
28	rs117767867	*SLC16A11*	0.13	0.13	1.06 (0.81–1.40)	0.67	1.06 (0.78–1.44)	0.71

OR: odds ratio; 95% CI: 95% confidence interval; * Adjusted for age, sex, body mass index (BMI), triglycerides (TG), total cholesterol (TC), low-density lipoprotein cholesterol (LDL-C), systolic blood pressure (SBP), and diastolic blood pressure (DBP).

**Table 3:** Allelic and genotypic distributions of rs17106184 in the healthy controls and T2DM patients.

rs17106184	Healthy Controls (*N* = 419)	T2DM Patients (*N* = 460)	Crude Model		Adjusted Model *	
			OR (95% CI)	*p*	OR (95% CI) *	*p*
Allele (%)						
G	784 (93.6)	813 (88.3)	Ref.		Ref.	
A	54 (6.4)	107 (11.7)	1.91 (1.36–2.69)	<0.0001	2.22 (1.53–3.24)	<0.0001
Genotype (%)						
GG	368 (87.8)	359 (78)	Ref.		Ref.	-
AG	48 (11.5)	95 (20.7)	2.03 (1.39–2.96)	<0.0001	2.30 (1.53–3.52)	<0.0001
AA	3 (0.7)	6 (1.3)	2.05 (0.51–7.36)	0.310	2.54 (0.70–11.03)	0.130
Dominant model (%)						
GG	368 (87.8)	359 (78)	Ref.		Ref.	
AA/AG	51 (12.2)	101 (22)	2.03 (1.41–2.93)	1.54 × 10^−4^	2.32 (1.57–3.47)	4.61 × 10^−5^
Recessive model (%)						
GG/AG	416 (99.3)	454 (98.7)	Ref.		Ref.	
AA	3 (0.7)	6 (1.3)	1.85 (0.46–7.46)	0.390	2.41 (0.63–10.12)	0.170
Additive model (%)						
GG:GA:AA	-	-	2.07 (1.34–3.02)	<0.0001	2.14 (1.47–3.12)	7.96 × 10^−5^

OR: odds ratio; 95% CI: 95% confidence interval; * Adjusted for age, sex, BMI, TG, TC, LDL-C, SBP, and DBP.

## Supplementary Materials

The following are available online at www.mdpi.com/1660-4601/13/9/863/s1, Figure S1. Linkage disequilibrium (LD) plot of IGF2BP2, CDKAL, 1C2CD4A/B and SLC16A11 (a) IGF2BP2; (b) CDKAL1; (c) C2CD4A/B; (d) SLC16A11; Figure S2: Linkage disequilibrium (LD) plot of a 100-kb 1p33 region based on HapMap-CHB data.
