# Supplementary material for: Validation of Type 2 Diabetes Risk Variants Identified by Genome-Wide Association Studies in Northern Han Chinese
Source: Int J Environ Res Public Health. 2016 Aug 30;13(9):863. doi: 10.3390/ijerph13090863 (PMC5036696; doi:10.3390/ijerph13090863)
Supplement: Supplementary file 1 [file ijerph-13-00863-s001.pdf]

Supplementary Materials: Validation of Type 2 Diabetes Risk Variants Identified by Genome-Wide Association Studies in Northern Han Chinese

Ping Rao, Yong Zhou, Si-Qi Ge, An-Xin Wang, Xin-Wei Yu, Mohamed Ali Alzain, Andrea Katherine Veronica, Jing Qiu, Man-Shu Song, Jie Zhang, Hao Wang, Hong-Hong Fang, Qing Gao, You-Xin Wang and Wei Wang

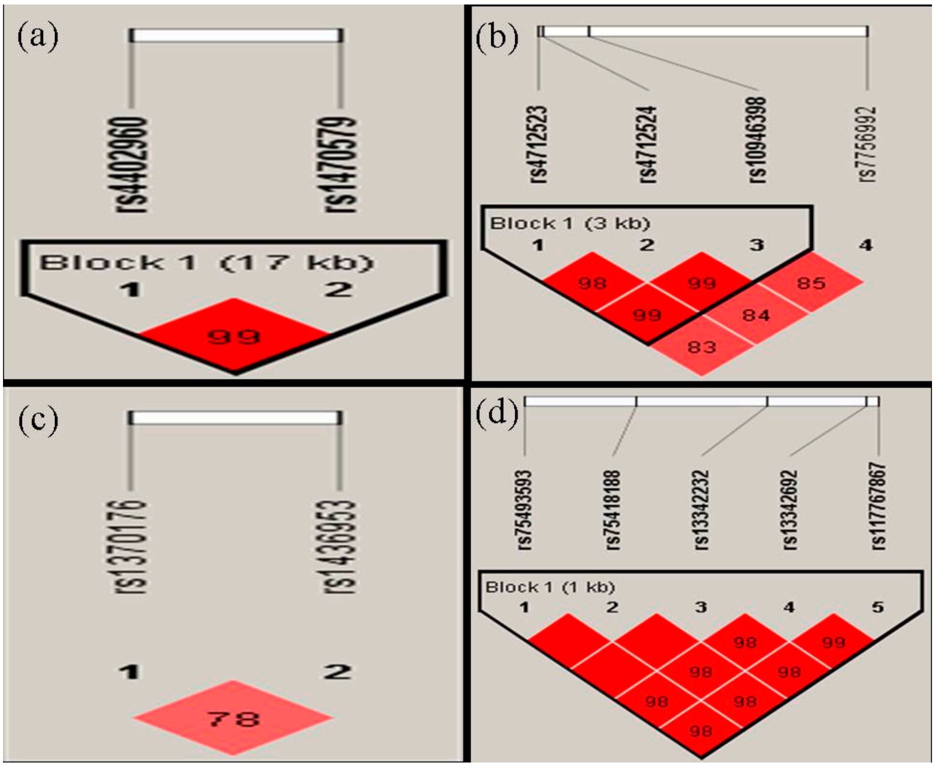

Figure S1. Linkage disequilibrium (LD) plot of IGF2BP2, CDKAL1, C2CD4A/B and SLC16A11 (**a**) IGF2BP2; (**b**) CDKAL1; (**c**) C2CD4A/B; (**d**) SLC16A11.

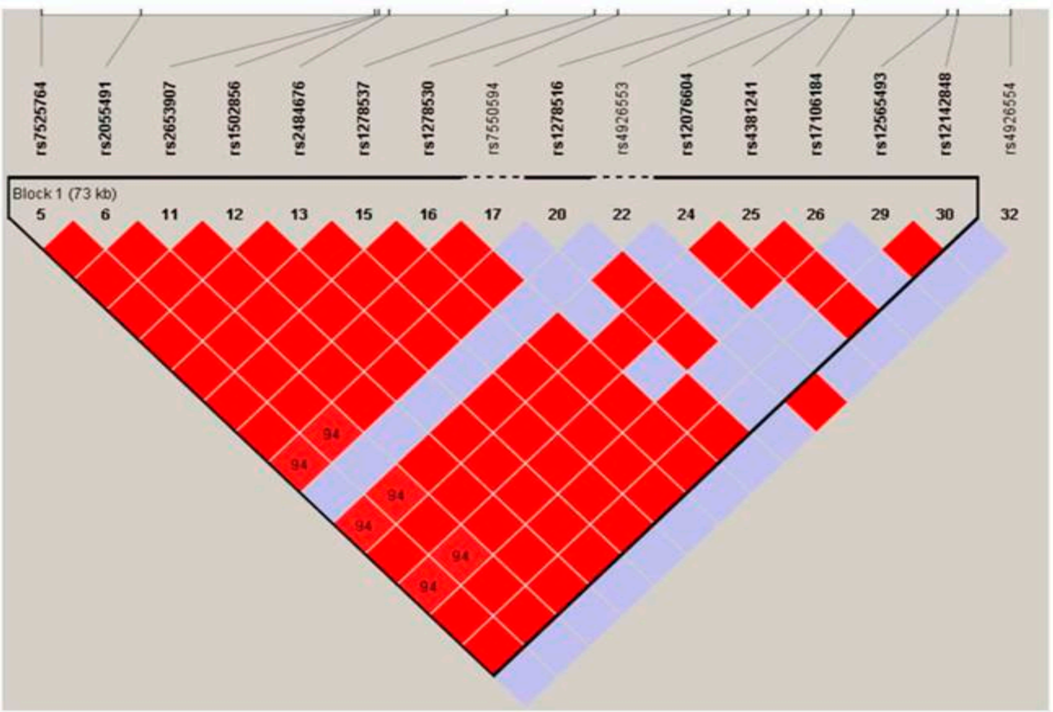

Figure S2. Linkage disequilibrium (LD) plot of a 100 kb 1p33 region based on HapMap-CHB data.

© 2016 by the authors. Submitted for possible open access publication under the terms and conditions of the Creative Commons Attribution (CC-BY) license (http://creativecommons.org/licenses/by/4.0/).
